# Exploring the role of electric vehicles in Africa's energy transition: A Nigerian case study

**DOI:** 10.1016/j.isci.2022.103926

**Published:** 2022-02-14

**Authors:** Michael O. Dioha, Lei Duan, Tyler H. Ruggles, Sara Bellocchi, Ken Caldeira

**Affiliations:** 1 Department of Global Ecology, Carnegie Institution for Science, Stanford, CA 94305, USA; 2 Department of Industrial Engineering, University of Rome Tor Vergata, Via Del Politecnico 1, I-00133 Rome, Italy; 3 Breakthrough Energy, Kirkland, WA 98033, USA

**Keywords:** Energy policy, Energy sustainability, Energy Resources, Energy transportation, Energy storage

## Abstract

We employed a bottom-up modeling framework to examine a set of scenarios to generate insights on the techno-economic and environmental implications of increasing levels of electric vehicle (EV) penetration using Nigeria as a case study. Results indicate that, despite Nigeria having a natural gas-dominated electricity system, the deployment of EVs can support the decarbonization of the transportation and power sectors but at a relatively high cost. The cost of EVs would need to drop by ∼40% to become cost-competitive. However, if variable renewable energy sources deliver the EVs power requirement with a bidirectional vehicle-to-grid (V2G) charging strategy, then the cost of EVs would need to decline by only ∼30%. Not all EVs need to participate in a V2G charging strategy in order to realize the full benefits of the strategy. Expanding renewables capacity leads to additional reduction in CO_2_ emission and decarbonization cost but at different magnitudes based on the charging strategy.

## Highlights

• Deploying EVs in Nigeria could support decarbonization
• V2G charging of EVs lessens pressure on Nigeria’s energy sector investments
• High EVs penetration is cost-effective with variable renewable electricity
• Benefit from V2G is achieved at relatively modest deployment levels of EVs

## Graphical abstract

**Figure:**
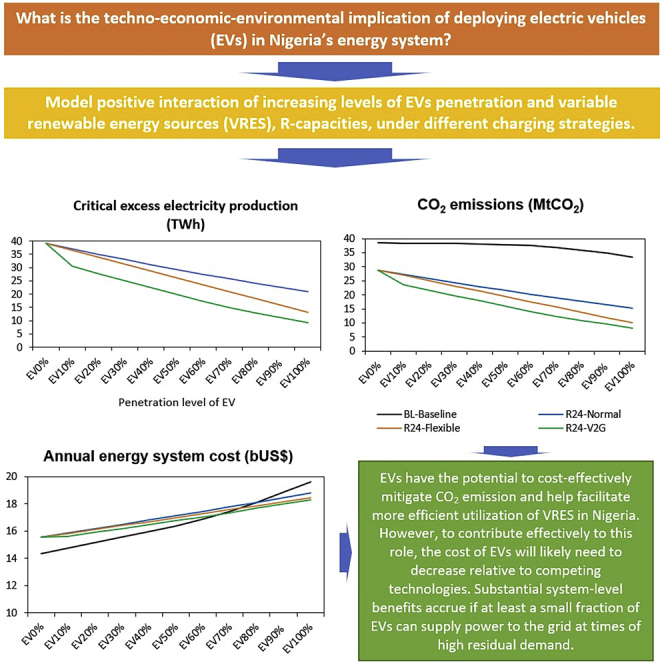

## Introduction

Many African countries will be making huge investments in the power sector in the coming years to expand energy access while promoting environmental sustainability (Ayamolowo et al., 2022; Du et al., 2021; Maji et al., 2019; Mutombo and Numbi, 2019; Qudrat-Ullah and Nevo, 2021). Also, vehicle ownership is expected to rise exponentially in the continent owing to the projected population growth rate, urbanization, and the general desire for private car ownership. Thus, African countries have the opportunity to pursue a sustainable and low-cost energy pathway by taking advantage of the current global electric vehicles (EV) momentum, while avoiding locking themselves into carbon-intensive energy systems (Hill et al., 2018).

Taking a closer look at a true representation of Africa, Nigeria is the most populous country in Africa, and Nigeria's vehicle fleet accounts for around 8.4% of the total vehicles in use in the continent (Deloitte, 2016). Nigeria’s rapidly growing population continues to strain existing infrastructure, transportation and energy, in particular (Dioha et al., 2019). Nigeria's transportation sector is powered almost entirely by fossil-derived gasoline and diesel, which is incompatible with climate mitigation actions from the long-term perspective (Dioha et al., 2021). This transport system also creates a cost burden for Nigerians and a fiscal burden for the government. The federal government of Nigeria (FGN) heavily subsidizes transport fuels, especially gasoline. The FGN spends around US$3.9 billion on fuel subsidies, which, is almost double the health budget and 2% of the gross domestic product (GDP) (McCulloch et al., 2021). As Nigerian cities expand, the demand for oil will increase, resulting in increased fuel scarcity, cost burden problems, and environmental pollution (Dioha and Kumar, 2020a). Meanwhile, the demand for electricity in Nigeria is rapidly increasing and it is expected to grow more than 10-fold by 2050 under a business-as-usual case (ECN, 2015). Consequently, Nigeria is looking towards low-cost renewables and has established policies to tap into its abundant renewable resources to grow electricity generation (FGN, 2016). Although electricity generation from variable renewable energy sources (VRES) often does not match the demand from consumers, energy storage that allows electricity to be saved and used at different times could enable the viability of renewables in Nigeria.

Nigeria could greatly benefit from three advances: a transport alternative to reduce the growing burden of fossil fuel dependency and expensive subsidies, a transport alternative to reduce environmental pollution, and an electricity storage solution to leverage its abundant VRES that can support decarbonization efforts. EVs can support these three advances simultaneously. A detailed description of EV technologies and their associated components are provided in Un-Noor et al. (2017). VRES and EVs can complement each other—EVs have the capability to provide storage for the excess electricity generated by VRES, which otherwise would be curtailed (Kühnbach et al., 2021). EVs with a vehicle-to-grid (V2G) system can also be used to supply energy to the grid at peak energy demand periods, while VRES could serve as a low-cost and carbon-neutral way for charging EVs (Deng et al., 2022; Himabindu et al., 2021; Lander et al., 2021). However, this interaction of EVs and the electricity supply system will depend on the charging strategy (Hanemann et al., 2017). When EVs are charged exclusively as per the drivers' charging habits without regard to the balance of generation and demand, it may worsen peak electricity demand. However, if EVs are charged only during low power demand or during excess electricity production, it may help to reduce grid overloading (Bellocchi et al., 2019). In this context, EVs may benefit Nigeria, but a sound knowledge of its implications on the Nigerian energy system is needed to insure their smooth deployment in the country.

In the literature, many studies have investigated the role of EVs in the strategic planning of the energy system (Liu et al., 2015). At a country (Bellocchi et al., 2018) and local level (Buonomano et al., 2019), EV charging strategy is projected to significantly impact the capability of EVs to serve as a demand management option. Other studies have examined the combination of EVs and residential heating to support VRES penetration (Bellocchi et al., 2020), alternative optimal future scenarios to understand the techno-economic implications of different penetration levels of EVs and VRES (Prina et al., 2019), life cycle emissions of EV charging strategy (Tang et al., 2021), incentive systems incorporating cryptocurrency for EV users (Zhang et al., 2018), the role of social class and behavior in EV commercialization (Lee and Brown, 2021), the influence of EVs on household electricity bills (Kühnbach et al., 2020), and policy recommendations to increase the uptake of EVs (Adoba and Dioha, 2021; Greene et al., 2014). The energy system impacts of EVs is well researched, and a detailed review and analysis of this subject matter are presented in Liu et al., 2015; Muratori et al., 2021; Nour et al., 2020; Taljegard et al., 2019. A key observation from this literature is that most studies have focused on the developed world.

There is no single answer for how EVs will impact the energy system because the impact of EVs varies substantially depending on factors such as the composition of the electricity generation mix. Only a handful of studies have focused on African countries (e.g., Nigeria) and the peculiarities regarding EV development in these countries. Here, we present a snapshot of these studies. A study on the environmental impact of EVs in South Africa suggested that the deployment of EVs could increase CO_2_ emission and other local air pollutants, as the country’s electricity supply mix is dominated by relatively low-quality coal (Liu et al., 2012). In a different context, the suitability of V2G technology was analyzed for Morocco (Ben Sassi et al., 2021).

Other studies related to EV prospects in Africa have explored the need for a more systemic approach to data collection to enhance EV studies in Africa (Collett and Hirmer, 2021); the importance of designing EV systems that are context-specific for Africa (Collett et al., 2021); electricity demand implications of solar-charging urban minibus EVs in South Africa (Abraham et al., 2021); grid impact of electrifying minibus taxis in Kampala, Uganda (Booysen et al., 2022); the feasibility of car park owners to provide solar PV carports for EV charging in South Africa (Buresh et al., 2020); and the potential minibuses in Cairo, Nairobi, and Cape Town toward promoting environmental sustainability (Odhiambo et al., 2021). An overarching analysis on the nexus between electrification and renewable energy deployment in developing countries proposed EV integration to maximize the use of excess electricity production from VRES (Bamisile et al., 2021). In terms of economic feasibility, a study concluded that owning an EV will cost 13.5% more compared with conventional gasoline-fueled vehicles in Ghana (Ayetor et al., 2020).

The preceding studies elucidate the advancement of the knowledge frontier in EV research from the perspective of African countries. Here, we set out to further advance this frontier. We introduce a model-based study on the positive interaction of EVs with the electricity supply system, examining the techno-economic-environmental implication of EVs in Nigeria within an integrated energy system framework. We define a baseline case for the Nigerian energy system calibrated with 2015 energy system data (see STAR Methods for further detail). We parametrically develop different EV penetration levels and VRES capacity relative to the 2015 baseline levels. These alternative energy system configurations are simulated with the help of a bottom-up modeling tool (EnergyPLAN) to examine the implications of increasing EV penetration and total installed VRES capacity. Our study also considers human behavior by showing how the energy system will respond to consumers' behavior toward a V2G bidirectional charging strategy. Results are assessed in terms of critical excess electricity generation from VRES, CO_2_ emission, and energy system cost for different charging strategies. We do not intend to predict/forecast the future sales of EVs or the evolution of Nigeria’s energy system; a corollary and more important objective is to provide a high-level analysis that can inform discussions on EVs deployment in Nigeria.

## Results

The variety of scenarios described (see STAR Methods details) have been examined with respect to the following indicators: excess electricity production from VRES, CO_2_ emission, and annual energy system cost. We focus our results and discussion on the maximum VRES capacity (R24) scenarios. We introduce results for R6, R12, and R18 clusters of scenarios when doing so helps to elucidate an important point.

### Critical excess electricity production from VRES

**Figure 1:**
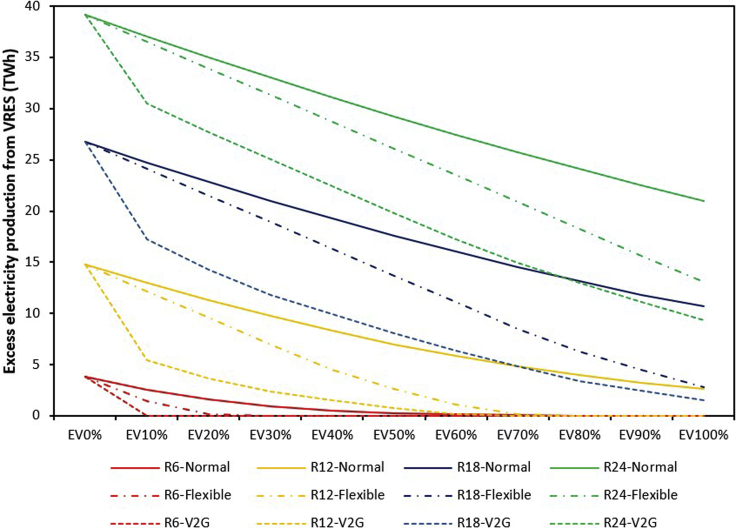
Excess electricity generation for increasing VRES capacity and EV penetration levels under different charging strategies. As VRES capacity is parametrically increased from R6 (6 GW) to R24 (24 GW), the amount of excess electricity production increases but shows a declining trend as the penetration of EVs increases across all VRES capacity levels. The rate of decline is determined by the charging strategy with V2G strategy showing the greatest ability to utilize VRES generation.

EVs can potentially utilize excess generation in high amounts of VRES capacity systems. With increased deployment of VRES power, times when wind and solar generation exceeds demand typically become more common, and there is critical excess electricity production. Figure 1 illustrates the amount of excess electricity production for the different levels of VRES penetration. Owing to VRES capacity's unavailability in the baseline, EVs integration has no impact on excess electricity production. However, as VRES capacity is parametrically increased from R6 to R24, the amount of excess electricity production increases, but shows a declining trend as the penetration of EVs grows across all VRES capacity levels. As EV penetration increases from 0% to 100%, our analysis suggests that curtailed electricity will decline by 46% for the maximum VRES (R24) capacity under normal charging. This declining trend of excess electricity production is due to the additional energy demand induced by the growing level of EVs.

Figure 1 shows that the capability of EVs to reduce excess electricity production from increasing VRES and thus serve as a flexible load/storage medium will depend on the charging strategy adopted. As EVs penetration increases from 0% to 100% at the maximum VRES capacity considered in this study (R24), excess electricity production falls by 67% and 76% under flexible and V2G charging strategy, respectively. This implies that the flexible and V2G charging strategy improves the value of EVs in mitigating critical excess electricity production by 21% and 30% points, respectively, compared with the normal charging strategy.

**Figure 2:**
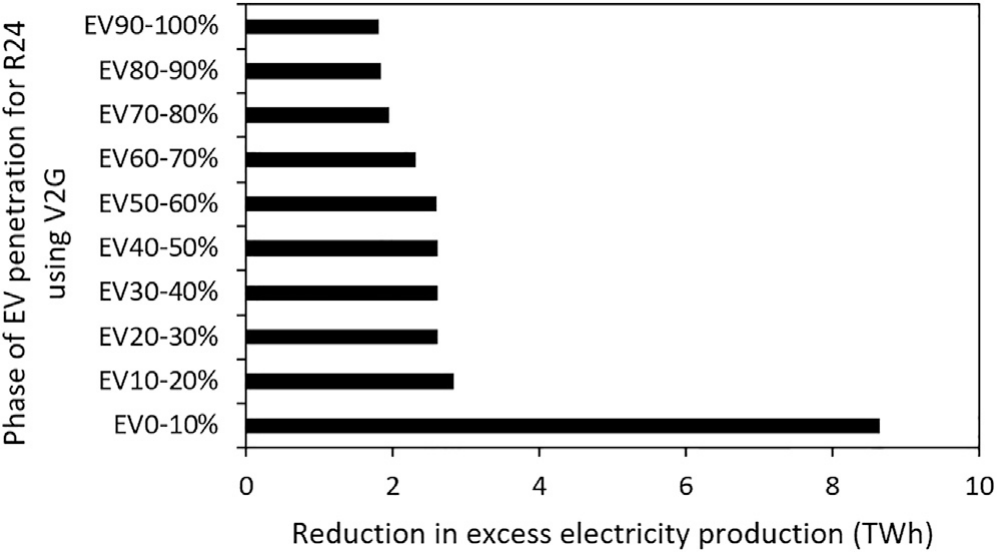
Reduction in excess electricity production at each phase of EV penetration for the maximum VRES capacity scenario (R24) using the V2G charging strategy. The largest incremental reduction is seen from the initial addition of EVs using a V2G strategy

Our analysis also shows that the value of V2G charging toward reducing excess electricity production from VRES is maximum at the initial phase of transition (between EV0 and EV10%). Initially, the introduction of V2G EVs that can supply battery power to the grid reduces peak residual demand and thus the need to build additional generation capacity. However, as EV penetration capacity increases, and thus also overall electricity demand, further introduction of EVs can require expansion of generation capacity. Therefore, most of the benefit from bidirectional V2G charging of EVs is achieved at relatively modest deployment levels. This result indicates that not all vehicles will be needed to participate in V2G charging to derive its substantial benefit. A combination of 10% EV V2G and 90% EV flexible charging may be enough to satisfy the power system's needs when EVs are integrated. To derive a deeper understanding of the result, we examine the changes (reduction of excess electricity production) that occur at each EV penetration rate in V2G charging mode (Figure 2). From Figure 2, it can be observed that the highest reduction of critical excess electricity production (∼9 TWh) occurs between 0% and 10% EV penetration rate.

### CO_2_ emission

**Figure 3:**
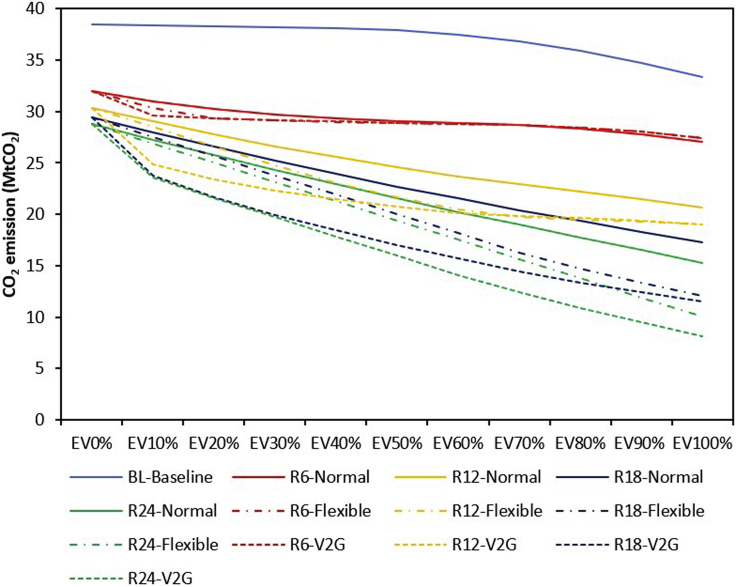
CO_2_ emission (transportation and power sectors) for baseline and increasing VRES capacity and EV penetration levels under different charging strategies CO_2_ emission drops gradually as the penetration level of EVs increases in the baseline case and drops further at different magnitudes as VRES capacity is parametrically increased from R6 (6 GW) to R24 (24 GW). However, the rate of decline is impacted by the charging strategy with V2G strategy showing the greatest ability to mitigate CO_2_ emission

EVs have the potential to reduce transportation sector-related emissions due to the absence of tailpipe emissions. Figure 3 illustrates the impact of EVs on CO_2_ emission. In the baseline power system without VRES capacity, CO_2_ emission drops gradually as the penetration level of EVs increases. Our analysis indicates that CO_2_ emission will decline by 13% in the baseline case, as EV penetration increases from 0% to 100%.

However, as VRES progresses from R6 to R24 under normal charging, CO_2_ emission reduces as EV penetration increases, but the rate of CO_2_ emission mitigation decreases and approaches its saturation level at R24. At maximum VRES (R24) capacity in our model, as EV penetration increases from 0% to 100%, CO_2_ emission drops by 47%. The results, therefore, indicate that the baseline power system supports the decarbonization of the Nigerian transportation sector. However, if the government desires to achieve deep decarbonization based on EVs, then it is pertinent to expand VRES capacity in the electricity generation mix. It may also be observed that there remains CO_2_ emission even at 100% EV deployment. This is because only the light-duty vehicles (LDVs) have been considered in this study, and so the transportation sector cannot be fully decarbonized unless the heavy-duty vehicles (HDVs) are also run by zero-emission vehicles/fuels.

The charging strategy affects the capability of EVs to mitigate CO_2_ emission. Figure 3 shows that, at maximum VRES capacity (R24) considered in this study, if EV penetration increases from 0% to 100%, CO_2_ emission is expected to drop by 65% and 72% under flexible and V2G charging, respectively. This implies that flexible and V2G charging leads to additional 18% and 25% points increase in CO_2_ mitigation compared with the normal charging strategy. V2G charging delivers the maximum CO_2_ mitigation benefit because during peak electricity demand, instead of sourcing power from natural gas-dominated conventional power plants, it uses electricity supplied by V2G charging vehicles to satisfy peak demand. This, in turn, decreases CO_2_ emission. Although V2G charging benefits are substantial, Figure 3 shows that the largest CO_2_ mitigation benefit of V2G charging is derived between EV0 to EV10%. This implies that the marginal benefit of V2G charging is maximum at the initial phase of transition.

**Figure 4:**
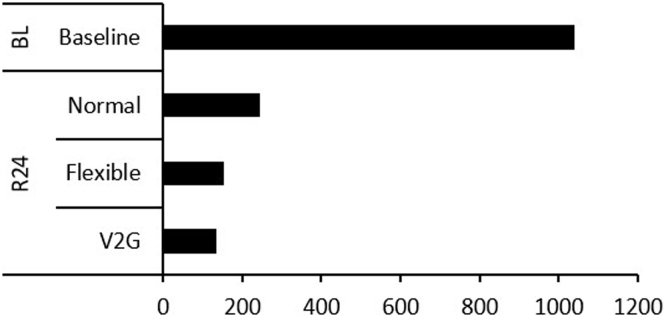
Cost per ton of CO_2_ emission mitigation (transportation and power sectors) at EV100% for baseline and maximum VRES capacity (R24) under different charging schemes. If EVs are charging based on natural gas (baseline), the cost of CO_2_ mitigation will be relatively high compared to when they are charged with V2G strategy in a renewbles based system.

Figure 4 shows model results for the unit cost of CO_2_ emission mitigation (i.e., change in total annual cost divided by change in CO_2_ emission) at 100% EV penetration for the baseline and VRES capacities (Figure 4). When EVs entirely replace LDVs in the baseline case, the model projects a cost per ton of CO_2_ mitigated is 1,041 US$/t. However, as VRES capacity is expanded, the cost per ton of CO_2_ avoided declines. At maximum VRES capacity (R24), the cost per ton of CO_2_ avoided is 243 US$/t for normal, 154 US$/t for flexible, and 134 US$/t for V2G charging strategies. This result indicates over three times reduction in the unit cost of CO_2_ mitigation in Nigeria by using EVs, if the normal charging power is sourced from renewables, and about seven times if the EVs participate in a V2G charging strategy.

### Annualized energy system cost

**Figure 5:**
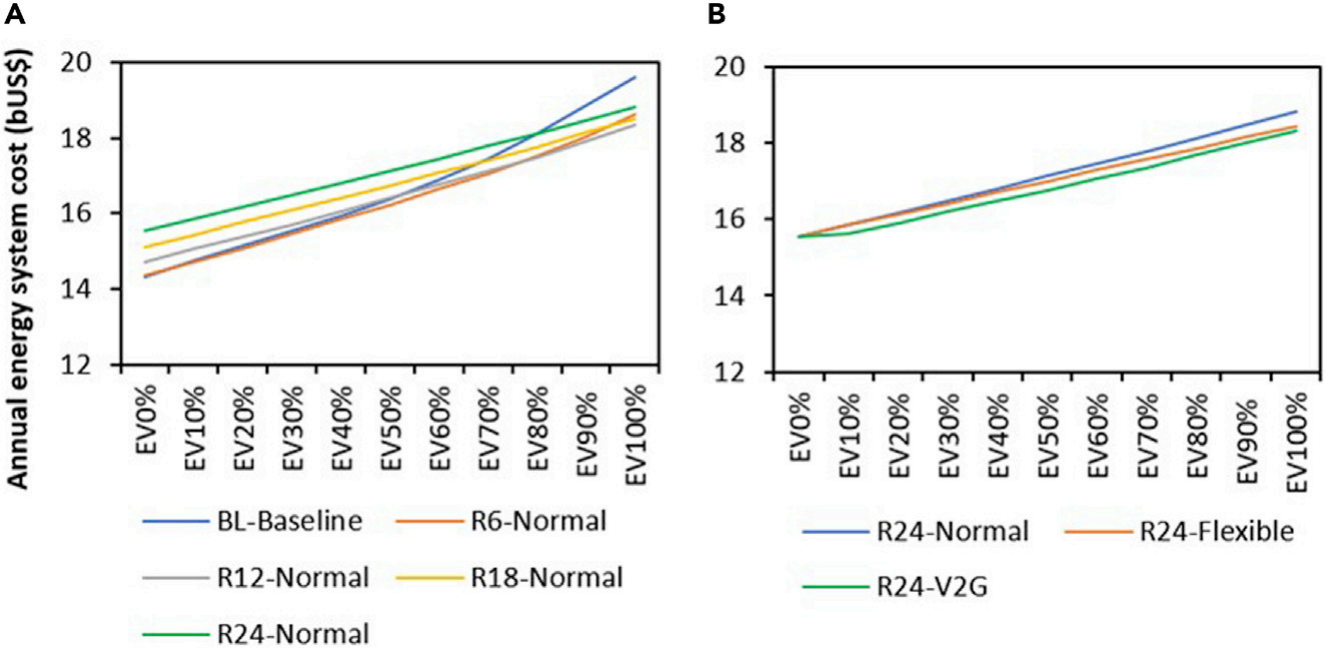
Energy system cost (transportation and power sectors) (A) Energy system cost for baseline and R6-R24 scenarios under normal charging. Energy systems with large VRES capacity are cost-effective at a high EV penetration levels compared to the baseline scenario. (B) Energy system cost at maximum VRES capacity (R24) under different charging strategies. The V2G charging strategy is cost-effective, and the impact is largest between EV0 and EV10% penetration

Figure 5 presents the total annual cost of the energy (transportation and power) system under a normal charging strategy for different VRES capacities and EV levels. The system’s total annualized cost increases almost linearly as the penetration level of EVs increases, irrespective of VRES capacity. Relative to 0% EVs, the total energy system cost increases by 37% at 100% EV penetration in the baseline case. However, if VRES powers the supply side as per R24-Normal, the total energy system cost increases by only 21% at 100% EV penetration. Figure 5 shows that at 10% EV penetration in R6 VRES capacity, the energy system cost becomes lower compared with the baseline case. However, at R24 VRES capacity, the energy system costs become lower than the baseline cost between 80% and 90% of EV penetration. Thus, the higher the VRES capacity, the longer it takes for the energy system cost to become lower than the baseline case. However, the metric of energy system cost may not capture all potential benefits of EVs.

Although the energy system cost is relatively low with R6 VRES capacity, the CO_2_ mitigation potential of EVs at R6 is also relatively low (Figure 3), whereas that of R24 is relatively high but comes at a higher energy system cost (Figure 5). As anticipated, Figure 5B shows that the charging strategy impacts the total energy system cost. Our analysis reveals that the energy system cost under flexible (R24-Flexible) and V2G charging (R24-V2G) grows by 19% and 18%, respectively, at 100% EV penetration. This is equivalent to 2% and 3% points gain, respectively, compared with the normal charging strategy. Therefore, the large-scale deployment of EVs will warrant additional costs on the supply side, whereas implementing a V2G charging strategy can reduce the pressure on upscaling energy system investments.

**Figure 6:**
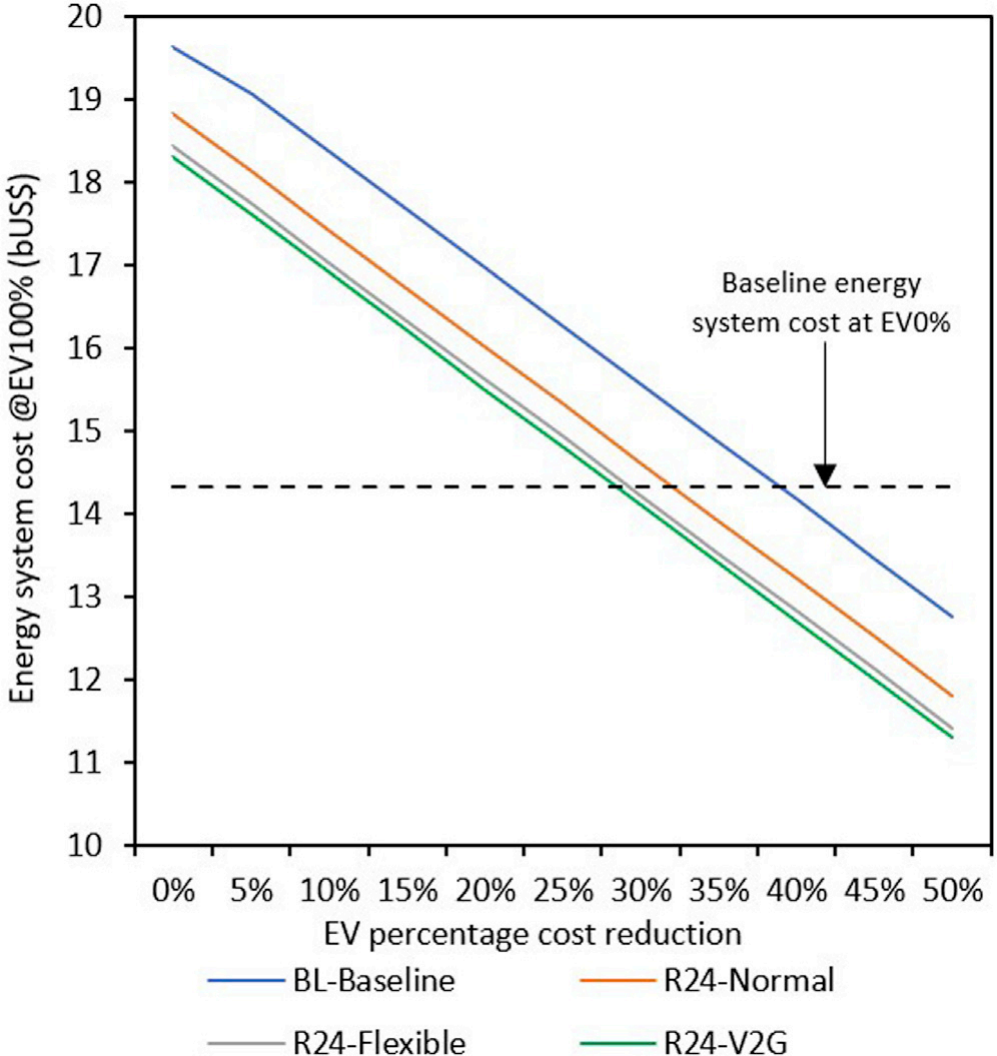
Effect of EV cost reduction on energy system cost (transportation and power sectors) at 100% EV penetration level. The cost of EVs would need to drop by ∼40% to become cost-competitive with the baseline case. However, if VRES deliver the EVs power requirement with a V2G charging strategy, then the cost of EVs would need to decline by ∼30%

From Figure 5, it was observed that completely replacing conventional vehicles with EVs is not a cost-effective option as the energy system cost increases compared with the existing system, irrespective of the VRES installation level. However, as EV technologies continue to mature, it is expected that the cost of EVs will drop in the future. Consequently, we examine at what cost EVs would be a cost-effective option relative to the existing system. Figure 6 shows that, in the baseline case where the energy system cost was US$14.33 billion and the gas-dominated power system supplies electricity for EVs, the cost of EVs needs to drop by around 40% before it can become cost-competitive. However, if the EVs power requirement is delivered by VRES (R24) under normal charging strategy, then the cost of EVs needs to decline by around 35% relative to competing technologies to become cost-competitive. Our analysis suggests that if a V2G charging strategy is employed, then the cost needs to fall by only around 30% to be cost-competitive. The lower cost reduction needed when EV power is from VRES is due to the absence of fuel cost as the power is delivered by solar and wind, unlike the baseline case where the power is delivered by natural gas.

**Figure 7:**
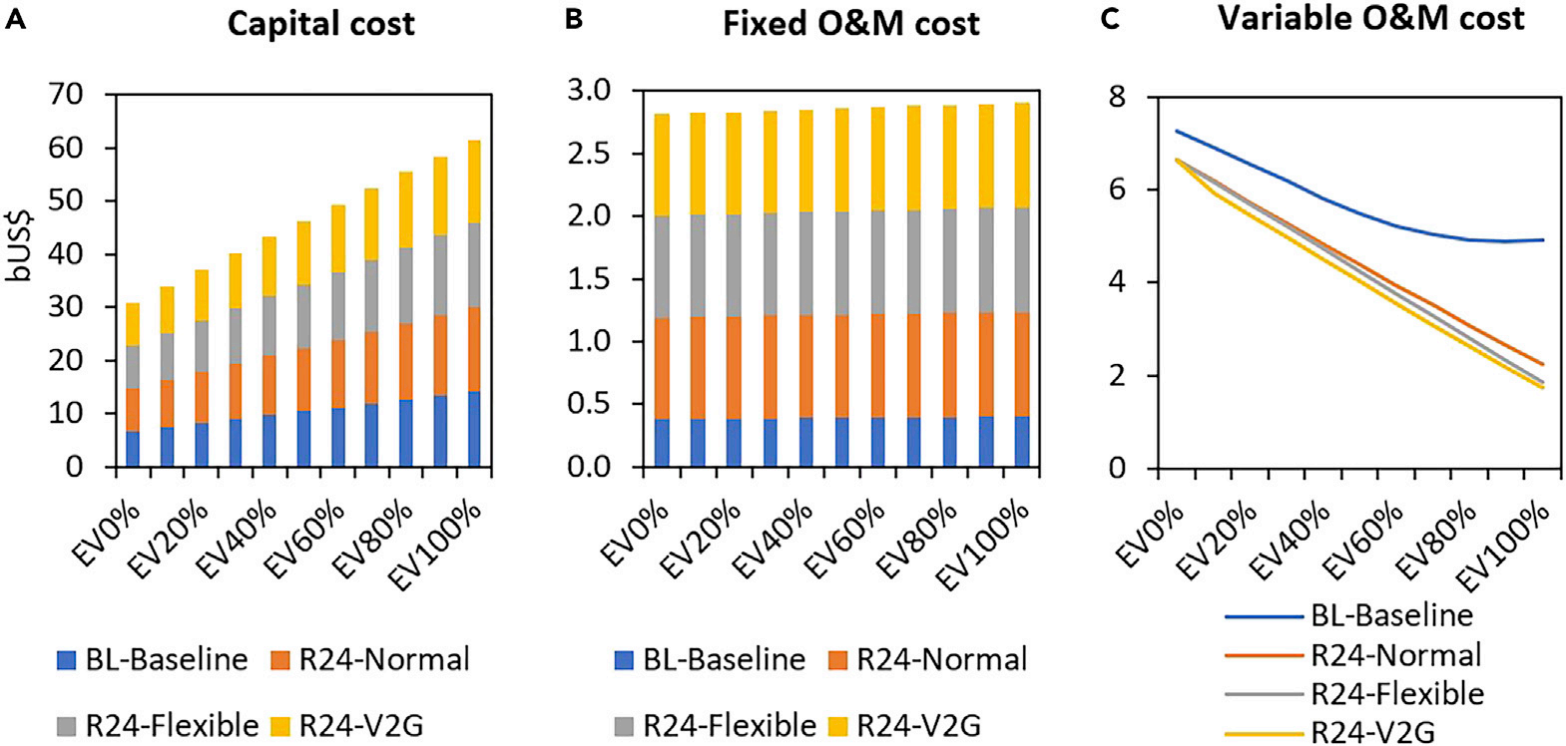
Disaggregated energy system (transportation and power sectors) cost (A–C) (A) Capital cost, (B) fixed O&M cost, and (C) variable O&M cost at baseline and maximum VRES (R24) capacity under different charging strategies

We have disaggregated the energy system costs into capital, fixed operation and maintenance (O&M), and variable O&M costs for the baseline and maximum VRES (R24) capacity under the different charging strategies (Figure 7). The capital cost (Figure 7A) grows linearly as the EV penetration level increases in the baseline and R24 scenarios but at different magnitudes. The growth trajectory observed in capital cost requirement is on account of the relatively higher cost of EVs compared with conventional vehicles. Similarly, in Figure 7B, we show that fixed O&M cost gently increases at higher EV penetration levels and it is also not impacted by the charging strategy. For the variable O&M cost (Figure 7C), a different outlook is observed on account of the reduction in fuel cost at greater EV penetration levels, which partly offset the total annual energy system cost. Variable O&M cost declines as EV penetration level increases, and the charging strategy impacts the variable cost of the energy system. As EV penetration increases from 0% to 100% in the baseline case, variable cost drops by 32.5%. However, if the system power is supplied by VRES (R24), the variable cost is further reduced to 66%, 725, and 74% under normal, flexible, and V2G EV charging strategies, respectively.

From Equation (3) (see STAR Methods details), the share of parked vehicles grid-connected is assumed to be 70%. However, energy systems are dynamic and sociotechnical systems, in which their evolution largely depends on human components of the system (Edomah et al., 2020). In reality, not all EV owners will be willing to charge their vehicles during excess electricity production from VRES or be willing to sell their battery power to the grid at a stipulated time owing to several reasons such as range anxiety (Geske and Schumann, 2018). As an experiment, we investigate a case where only 10% of the parked vehicles are grid-connected at maximum VRES (R24) capacity under a V2G charging strategy.

**Figure 8:**
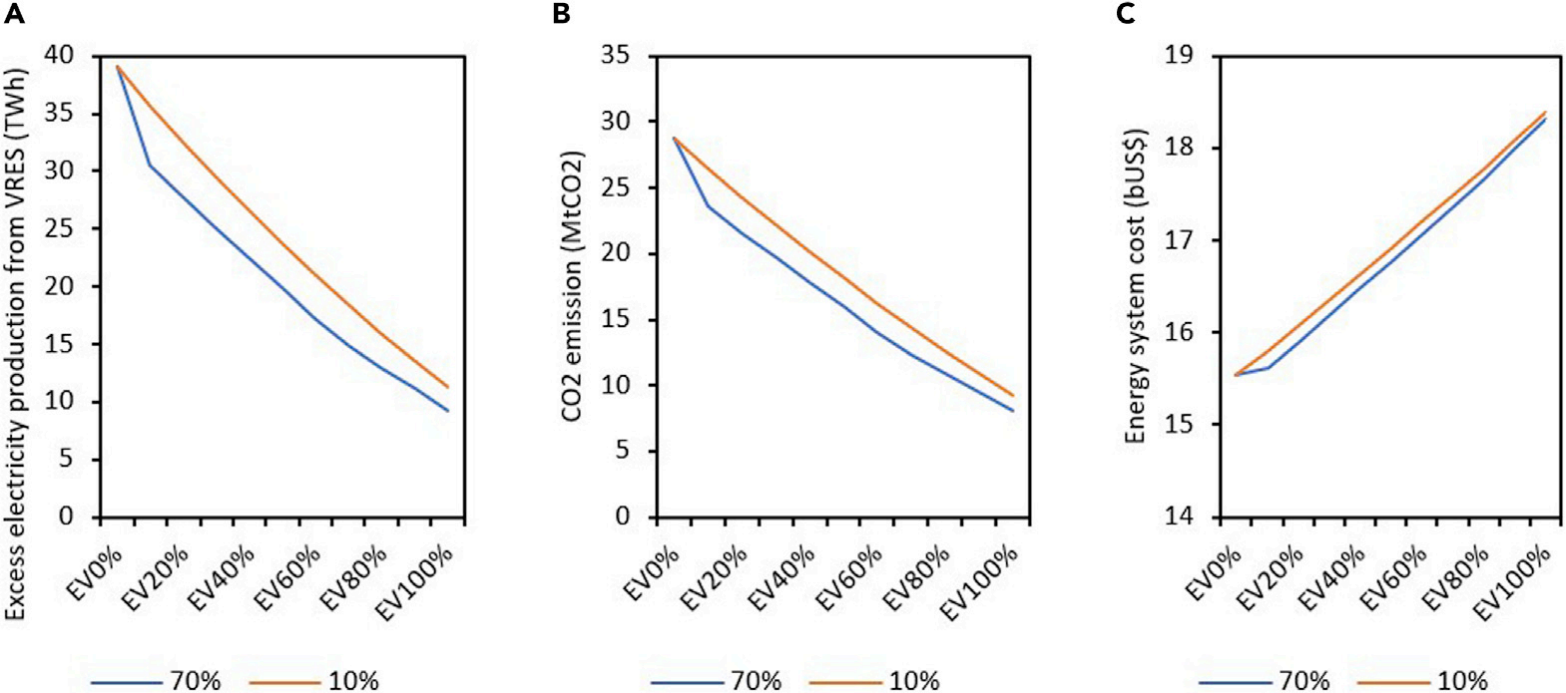
Variation of parameters when the share of parked grid-connected vehicles changes from 70% to 10% at maximum VRES (R24) capacity under V2G charging strategy (A–C) (A) Excess electricity production from VRES (TWh); (B) CO_2_ emission (MtCO_2_); (C) energy system cost (bUS$).

Figure 8 shows that excess electricity production, energy system cost, and CO_2_ emission increase when only 10% of the parked vehicles are grid-connected compared with the 70% case. Figure 8 indicates that the impact of shifting from 70% to 10% is more pronounced at lower EV penetration levels. An increase in EV penetration level reduces the effects of changing the share of parked vehicles on excess electricity production, energy system cost, and CO_2_ emission.

**Table 1:** Estimates of light-duty vehicles in Nigeria

Vehicle type	2010 Estimate (Cervigni et al., 2013)	2020 Estimate (Cervigni et al., 2013)	Calculated 2015 number (Source: Authors)
Motorcycle	3322888	7159073	5240981
Private car	4650509	8374026	6512268
LCV	557301	1319334	938318
Light Bus	5344	12650	8997

**Table 2:** Technological assumptions for light-duty vehicles in 2015

LDV type	Number of vehicles (2015)	Average annual utilization (km)	Average load factor	Average fuel economy(BPkm/PJ)^a^	Lifetime (years)	Battery capacity (kWh)
Motorcycles - Gasoline	5240981	7000	1.5	2.83	8	–
Motorcycles - Electric	0	7000	1.5	8.10	8	8
Private Car - Gasoline	6382022	17000	1.8	1.60	20	–
Private Car - Diesel	130245	17000	1.8	1.52	20	–
Private Car - Electric	0	17000	1.8	4.32	20	35
LCV - Gasoline	919551	33256	4.0	1.16	18	–
LCV -Diesel	18766	33256	4.0	1.10	18	–
LCV - Electric	0	33256	4.0	3.11	18	42
Light Bus - Gasoline	8817	33256	12.0	2.23	15	–
Light Bus - Diesel	180	30000	12.0	2.09	15	–
Light Bus - Electric	0	30000	12.0	4.03	15	90

a Billion passenger km per Petajoule.

**Table 3:** Assumptions on fuel prices

Technology	Price (US$/GJ)
Diesel	16.64
Gasoline	18.9
Natural gas	3.7

**Table 4:** Cost assumptions for vehicles

LDV type	Cost (US$/unit)	Comment
Motorcycles - Gasoline	960	Jincheng JC series is used as a proxy for motorcycles
Motorcycles - Electric	1500	The Indian eFTR Jr is used as a proxy for electric motorcycles
Private Car - Gasoline	18800	Private car includes different brands of saloon, wagon, and SUVs. Toyota corolla is used as a proxy for private gasoline cars
Private Car - Diesel	21616	Peugeot 407 is used as a proxy for private diesel cars
Private Car - Electric	43464	The rear-drive Tesla Model 3 is used as a proxy for private electric cars
LCV - Gasoline	18500	Average cost of gasoline Toyota corolla, Toyota Camry, and Toyota HiAce
LCV -Diesel	21116	The diesel Toyota HiAce is used as a proxy
LCV - Electric	43464	The rear-drive Tesla Model 3 is used as a representative for private electric cars. We assume that future light commercial vehicles (taxis) will be automated with the growing Uber and Bolt services. It is expected that the use of *danfos* will decline in the future
Light Bus - Gasoline	31867	The gasoline Toyota HiAce is used as a proxy
Light Bus - Diesel	39833	The diesel Toyota HiAce is used as a proxy
Light Bus - Electric	52805	KINGSTAR EJ6 is used as a proxy

**Table 5:** Assumptions on techno-economic parameters of power plants

Technology	Lifetime (years)	Efficiency	Capital (US$/kW)	Fixed O&M (% of investment)
Gas turbine	30	0.3	1054	2.5
Large hydro	50	1.0	3171	1.25
Solar PV	25	1.0	900	1.1
Wind	25	1.0	1700	3.4

**Table 6:** Different VRES capacity considered (GW)

Technology	BL	R6	R12	R18	R24
Solar PV	0	5	10	15	20
Wind	0	1	2	3	4
Total VRES capacity	0	6	12	18	24

**Table 7:** Energy consumption of light-duty vehicles at different EV penetration levels (TWh)

EV penetration level (%)		EV0%	EV50%	EV100%
Motorcycle	Gasoline	5.40	2.70	0.00
	Electric	0.00	0.94	1.89
Private Car	Gasoline	33.90	16.95	0.00
	Diesel	0.73	0.36	0.00
	Electric	0.00	6.41	12.81
LCV	Gasoline	29.83	14.91	0.00
	Diesel	0.06	0.03	0.00
	Electric	0.00	5.57	11.15
Light Bus	Gasoline	0.40	0.20	0.00
	Diesel	0.00	0.00	0.00
	Electric	0.00	0.11	0.22

**Table 8:** Scenarios description

S/No	Cluster of scenarios	Description
1	BLEV0% – BLEV100%	BL0EV0% refers to the baseline case where VRES capacity is 0 GW, and EV penetration is 0%. In this study, the baseline VRES capacity is kept constant, and EV penetration is increased and analyzed from 0% to 10, 20, 30, 40, 50, 60, 70, 80, 90, and 100%. BLEV100% refers to the baseline case of VRES with 100% EV in the LDV fleet. These parametric increases yield 11 scenarios in total
2	R6EV0% – R6EV100%	R1EV0% refers to the first case where total VRES capacity is 6 GW, and EV penetration is 0%. The same analytical approach used in cluster 1 scenarios is applied here
3	R12EV0% – R12EV100%	R12EV0% refers to the second case where total VRES capacity is 12 GW, and EV penetration is 0%. The same analytical approach used in cluster 1 scenarios is applied here
4	R18EV0% – R18EV100%	R18EV0% refers to the third case where total VRES capacity is 18 GW and EV penetration is 0%. The same analytical approach used in cluster 1 scenarios is applied here
5	R24EV0% – R24EV100%	R24EV0% refers to the fourth case where total VRES capacity is 24 GW, and EV penetration is 0%. The same analytical approach used in cluster 1 scenarios is applied here

## Discussion

As Nigeria moves to invest in VRES to satisfy its electricity deficit (FGN, 2016), this is the perfect time for the country to explore the potential of EVs in its electricity planning strategies. Variability of renewables remains one of the key challenges limiting the smooth integration of renewables such as solar and wind into the grid system (Tambari et al., 2020). Our analysis shows that EVs can contribute toward addressing this challenge for Nigeria. When the Nigerian LDVs fleet is completely electrified, our model result suggests that curtailed electricity could decline by 46% for the maximum VRES (24 GW) capacity considered under the normal charging strategy (Figure 1). This value further increases to 76% when V2G charging strategy is employed. Although this looks promising, for Nigeria to benefit from the potential benefit of EVs, the current grid needs to be expanded and upgraded to support the integration of VRES and EV simultaneously. Currently, the Nigerian transmission wheeling capacity is about 5.4 GW, which is far below the total installed generation capacity of about 12.5 GW (Akanonu, 2020). The entire transmission infrastructure is characterized by incessant system collapses, thus creating reliability issues. Integrating additional VRES capacity and bidirectional EVs into the transmission network calls for accelerated infrastructure upgrade (Agunbiade and Siyan, 2020). A similar argument was made for South Africa by Buresh et al. (2020), who suggest that the South African grid will need significant intervention to handle the large-scale deployment of EVs.

From Figure 1, we also found that just about 10% of the LDVs fleet is enough to derive the full benefits of bidirectional V2G EVs. If the HDVs are included, this value could be much lower. This implies that the Nigerian government can mandate only government and commercial vehicles to participate in bidirectional V2G charging without “disturbing” the peace of private EV owners who may not be willing to sell their EV power to the grid. The modest fraction of EV needed to reap the full benefits of bidirectional V2G EVs can be partly attributed to the nature of the Nigerian power system as it consists of centralized- and off-grid generation. Currently, over 75% of Nigeria’s power generation capacity is from off-grid systems (Roche et al., 2020). Our analysis assumed that only centralized systems charge the EVs (see STAR Methods details). Renewable energy-based off-grid systems are usually equipped with battery storage to provide reliable power (Nnaji et al., 2019) and, thus may not require V2G services. This scenario also shows the importance of contextually treating EVs' value in energy systems. This result is pertinent for Nigerian decision-makers and energy planners as it gives a sense of the level of V2G EVs needed in the power system to mitigate potential excess electricity production from different centralized VRES capacities that may be installed in the future.

As the number of fossil fuel-based vehicles continues to increase in Nigeria, it is expected that the resultant transportation-related local air pollution and CO_2_ emission will increase. Nigeria is not immune from the adverse health effects of air pollution. Millions of Nigerians living in traffic-congested cities such as Lagos, Owerri, and Port Harcourt suffer from respiratory and cardiovascular diseases due to exposure to polluted air (Odogun and Georgakis, 2019). In terms of climate action, Nigeria is a party to the United Nations Framework Convention on Climate Change and has pledged to reduce its emissions by 20% below business-as-usual by 2030, in line with the Paris Agreement (Dioha and Kumar, 2020b). Also, Nigeria’s President, Muhammadu Buhari, pledged net-zero emission for Nigeria by 2060 during the 2021 United Nations Climate Change Conference (COP26) in Glasgow (Lo, 2021). These climate pledges have now put Nigeria’s transportation sector in a carbon-constrained scenario.

Figure 3 shows that Nigeria can reduce its natural gas- and gasoline-dominated power and transportation sectors' CO_2_ emission by 13% with the full electrification of its LDVs. Similar findings were made for Switzerland, where the utility-scale gas power plants promote EV deployment to mitigate CO_2_ emission (Kannan and Hirschberg, 2016). It should be stressed that not all fossil-dominated utility systems support EV for CO_2_ mitigation, just as in South Africa, where EV increases CO_2_ emission due to the coal-dominated central utility (Liu et al., 2012). However, it is worth mentioning that a recent study on EVs potential in South Africa suggested that EVs could reduce CO_2_ emission in the future if the country taps into its abundant renewable energy resources for EV charging (Tongwane and Moeletsi, 2021). Our results further indicate that, if Nigeria expands its centralized VRES capacity to 24 GW and employs a bidirectional charging strategy, this value could increase to 72%. Financing Nigeria’s climate targets remains a major hurdle the government needs to cross (Dioha and Kumar, 2020b). Figure 4 shows that it is cost-effective to mitigate CO_2_ emission by using V2G charging strategy. This suggests that, if the Nigerian government wants to achieve higher CO_2_ and cost-effective mitigation potential by expanding VRES capacity, it should consider coupling VRES expansion with a V2G charging strategy to deliver maximum benefits.

Access to modern energy services continues to elude many households in Nigeria (Dioha et al., 2022). Currently, Nigeria has the largest energy access gap globally, with over 85 million people lacking grid electricity. One of the key obstacles limiting energy access in the country is the lack of adequate finances for the power sector (Dioha and Emodi, 2019). Our results (Figure 5) indicate that electrifying Nigeria’s LDVs under the current electricity generation mix could increase energy system costs by 37% because of the expansion of generating assets needed to power the EVs. This implies that EVs are not cost-effective for Nigeria under the current electricity mix. However, if the expansion of VRES transforms the current electricity mix, the annualized energy system costs become lower than the baseline cost between 80% and 90% of EV penetration level. This cost-effectiveness further increases if V2G-bidirectional charging EVs is employed.

Accordingly, the mitigation potential of EVs is greatest when EVs can promote the use of VRES instead of fossil fuels. This potential is greatest when there is substantial penetration of VRES, contributing to better air quality and improved energy security (Farzaneh et al., 2018). Monetizing these additional benefits can make EVs even more cost-effective beyond the present results. Therefore, it is pertinent to understand potential synergies and trade-offs among these parameters when analyzing the positive interaction of EVs and the electricity generation system. Overall, Figure 5 suggests that it is cost-effective to add VRES to the system at high EV penetration compared with the natural gas-dominated baseline system.

Under the current gas-dominated electricity mix, our analysis indicates that the cost of EVs needs to drop by 40% for it to be cost-effective (Figure 6). However, if the present system is transformed by introducing large VRES and supported by V2G charging strategy, then a 30% decrease in the cost of EVs may make it a cost-effective option with respect to the existing transportation and power system. In both cases (baseline and VRES), bringing down the cost of EVs would make electric mobility cost-effective compared with the existing system. This suggests that it will be difficult for EVs to penetrate the Nigerian vehicle market presently. This result underscores the need for further research and development to bring down the cost of EVs to a level that is competitive with its internal combustion engine vehicles counterparts to make it a cost-effective option. To reduce the cost of EVs, the Nigerian government may want to consider introducing robust fiscal incentives to reduce the upfront cost of EVs and support local automotive industries.

Our results show that, for the three metrics analyzed, excess electricity production from VRES, CO_2_ emission, and annual energy system cost, bidirectional V2G EVs prove to be the most beneficial. This result further highlights the importance of incorporating a V2G charging strategy into EV policy frameworks. However, as noted earlier, many EV owners may not be willing to sell their battery power to the grid at a stipulated time owing to several reasons (Geske and Schumann, 2018). Beyond range anxiety, one of the key issues that EV owners grapple with in bidirectional EVs is whether discharging their vehicle batteries to the grid will be detrimental to their EV batteries because of the additional cycling introduced. A recent study on this subject matter highlighted that ignoring battery degradation when canvassing for bidirectional EVs is not viable (Uddin et al., 2018). Dubarry et al. (2017) examined the battery (Li-ion) implication of bidirectional EV by selling as much capacity as possible during 1-h periods where the grid needs it the most. They found that additional usage of the batteries, even at constant power, is detrimental to cell performance and that it could shorten the lifetime of battery packs to less than 5 years. This implies that the Nigerian government should be willing to map out robust incentives for bidirectional EVs owners to reduce their fears, to reap the potential benefits that bidirectionality can offer.

## Conclusions

This study has examined the implications of increasing the penetration of EVs at different VRES capacities in Nigeria. The results have been analyzed in the context of critical excess electricity production, CO_2_ emission, and energy system cost. Our analysis shows that the introduction of EVs into the present Nigerian energy system will support decarbonization. However, if the power supply system is transformed by introducing large VRES, the introduction of EVs will lead to additional decarbonization compared with the baseline case. Indeed, the electricity generation mix plays a key role in the potential of EVs to reduce CO_2_ emission. The additional CO_2_ mitigation potential derived from expanding VRES capacity from 6 GW (case R6) to 24 GW (case R24) highlights the need for an integrated approach that couples EV deployment with VRES-based electricity generation.

As VRES capacity is expanded from 6 GW to 24 GW in the electricity generation system, the amount of critical excess electricity production increases, but shows a declining trend as EV deployment progresses. The capability of EVs to support the mitigation of excess electricity production strongly depends on the charging strategy. As EVs penetration increases from 0% to 100% at the maximum VRES capacity considered in this study (24 GW), excess electricity production falls by 67% and 76% under flexible and V2G charging strategy, respectively. However, our analysis shows that the marginal benefits of the V2G charging strategy is at a maximum at the initial introduction of V2G EVs and most of the benefits from V2G EVs (i.e., ability to provide power at times of peak residual demand) is achieved by the time a 10% V2G EV penetration level is reached. Therefore, not all vehicles need to participate in V2G to derive the full benefits of a V2G charging strategy.

As observed across the techno-economic-environmental indicators used in this study, the maximum relative benefits of EVs are derived when a substantial fraction of them participate in V2G charging. Hence, there may be benefits from an EV charging regulation that incentivizes EV owners to charge/discharge their vehicles based on the power network needs. However, in practice, this regulation will be challenging to enforce based on a social perspective as the charging/discharging time required by the power network may not always align with the transport requirements of the EV owners. Thus, incentives to enhance the propensity of EV owners to change their behavior and switch to a V2G charging strategy may yield substantial system-level benefits.

At present, the introduction of EVs is not a cost-effective option for Nigeria. The cost of EVs needs to drop by 40% to be cost-effective under the current energy system. However, if the present system is transformed by introducing large VRES and supported by V2G charging strategy, then a 30% decrease in the cost of EVs may make it a cost-effective option with respect to the existing transportation system.

Fundamentally, this study indicates that EVs have the potential to help facilitate more efficient utilization of variable renewable resources in Nigeria. However, to contribute effectively in this role, the cost of EVs will likely need to decrease relative to competing technologies. Substantial system-level benefits accrue if at least a small fraction of these vehicles can supply power to the grid at times of high residual demand. Policymakers and energy system planners working in Nigeria may want to consider the potential benefits of policies aimed at promoting the further deployment of EVs.

### Limitations of the study

The scope of this study is limited to the transportation and power sectors of Nigeria. The model employed in this study (EnergyPLAN) is designed to ensure that the EV battery is fully charged before it disconnects. In the real world, this may not be the case (for instance, EV owners would not like to wait for full charge to use their vehicles in an emergency). As a result, the value of EV in reducing critical excess electricity production, CO_2_ emission, and energy system cost may differ from the ones analyzed here. We assumed that traffic distribution is uniform in the country. In practice, significant variations exist in traffic distribution across different cities in Nigeria. A dedicated city-level survey would be needed to integrate these traffic distributions in future studies. Also, only tailpipe CO_2_ emission is considered in this study. EVs contain embodied emissions that may affect our results if considered. Infrastructure cost plays a role in assessing the cost-effectiveness of EV deployment. The study is based solely on the capital cost of EVs. In sum, we recognize that some uncertainties may affect our results, which opens the window for future studies on this subject matter. While acknowledging these uncertainties in our paper design, our results provide a sense of the level of efforts and policies needed to smoothly integrate EVs into the Nigerian vehicle fleet.

## STAR★Methods

### Key resources table

**Table:** 

REAGENT or RESOURCE	SOURCE	IDENTIFIER
**Software and Algorithms**		

EnergyPLAN	(Lund et al., 2021)	https://www.energyplan.eu/

**Assumptions on Technologies**		

Fuel economy of vehicles	(Dioha and Kumar, 2020a)	https://doi.org/10.1016/j.jup.2020.101034
Vehicle stock	(Cervigni et al., 2013)	https://doi.org/10.1596/978-0-8213-9973-6
Power plants	(Roche et al., 2020)	https://doi.org/10.1080/14693062.2019.1661818

**Cost Assumptions**		

Power plants	(Nigerian Economic Summit Group and Heinrich Böll Stiftung Nigeria, 2017)	https://ng.boell.org/sites/default/files/true_cost_of_power_technical_report_final.pdf
Vehicle cost	Multiple sources	https://www.ccarprice.com/ng/, https://carsforsalenigeria.com/, https://naijauto.com/, https://autochek.africa/ng, and https://carmart.ng/
Fuel cost	(Dioha and Kumar, 2020b)	https://doi.org/10.1016/j.enpol.2020.111703

### Resource availability

#### Lead contact

Further information and requests for resources should be directed to and will be fulfilled by the Lead Author, Dr. Michael O. Dioha (mdioha@carnegiescience.edu).

#### Materials availability

This study did not generate new materials.

### Method details

In the interest of clarity and analytical tractability, the analysis presented here is based on the assumptions that all EVs are battery electric vehicles (BEVs) and are charged by only the central electricity supply system; there is no available grid-storage for intermittent electricity production from VRES, and the dynamic response of the power system remains stable. It is also assumed that all vehicles and power plants are new installations.

#### Analytical framework

The core of this study is the techno-economic modeling of Nigeria's power and transportation energy system. Over the last three decades, the scientific community has developed a variety of energy modeling frameworks. These models vary in their analytical architecture and the type of research questions they answer. Energy models are typically classified as top-down or bottom-up based on their analytical approaches (Dioha, 2017). The top-down models are used to assess the macro-economic implications of energy system developments, but they do not represent technologies in detail. Typical examples of top-down models include the computable general equilibrium models (e.g., GEM-E3 and GTAP (Gargiulo and Gallachóir, 2013)) and macro-economic models (e.g., HERMES (Bergin et al., 2013) and NIGEM (NIGEM, 2021)). In contrast, the bottom-up models, otherwise known as the engineering-oriented models, are technology-rich models usually written as mathematical programming problems to examine the interactions of energy and technologies but lack detailed macro-economic feedback systems. Typical examples of bottom-up models include the integrated cost-optimization energy system models (e.g., MARKAL/TIMES and MESSAGE) and integrated energy system simulation models (e.g., LEAP and EnergyPLAN) (Bhattacharyya and Timilsina, 2010; Gargiulo and Gallachóir, 2013).

This study uses, primarily, a bottom-up modeling framework – EnergyPLAN (Version 15.1) – to define a simple model for the Nigerian central electricity supply system and its integration with the transportation sector for the year 2015, the most recent milestone year with sufficient data availability. The tool is freeware and has been used to study future sustainable energy systems, emphasizing the deployment of high shares of VRES in the energy mix. The tool is mainly applied in designing regional, national and local energy systems to examine the techno-economic-environmental implications of different energy choices. The main purpose of EnergyPLAN is to provide a transparent foundation for informed discussion on possible developmental pathways for an energy system instead of predicting/forecasting the evolution of an energy system (Lund et al., 2017). EnergyPLAN is a deterministic model that performs its analysis on an hourly basis for a single year, and the implications are analyzed in terms of various technical and market-economic simulation strategies.

The fundamental modeling logic in EnergyPLAN is known as "analytical programming". Unlike traditional optimization models, which seek to provide a cost-optimal energy mix by solving a series of equations, EnergyPLAN is designed based on a number of endogenous priorities embodied in a set of rules (Lund and Thellufsen, 2020). The EnergyPLAN approach is completely deterministic with no stochastic elements. Instead, various simulation strategies are used to determine the analytical operation of the modeled system. Typically, the model is used for technical, market exchange, and feasibility studies. This study focuses on the technical simulation strategy based on the least-fuel-consuming solution. Thus, this strategy prioritizes the utilization of VRES ahead of conventional power plants.

A distinguishing characteristic of EnergyPLAN is the “smart energy system” concept it uses to define an energy system configuration. A smart energy system is defined by 100% renewable energy, sustainable biomass consumption, efficiency maximization, and affordability. The concept of a smart energy system is based on futuristic technologies and is further described in (Lund et al., 2012; Mathiesen et al., 2015). EnergyPLAN can be used to simulate a complete energy system, not just the electricity system. EnergyPLAN can also be used for other sectoral analyses depending on the research question. The idea in EnergyPLAN is that an aspect of the energy system should not be treated in isolation if it might materially affect other sectors. For instance, because the deployment of EVs in the transportation sector could affect electricity system operation, there is motivation to analyze their impact in the context of an integrated energy system. This feature makes EnergyPLAN a useful tool for our study as it allows us to examine the interaction of the transportation sector and electricity supply system with high shares of VRES.

Several kinds of input parameters are needed to examine the energy balances of an energy system, simulating its operational function for a given year on an hourly mode. For our study, the demand side data includes the transportation sector's annual electricity demand and fuel consumption and their associated hourly distributions (Lund and Thellufsen, 2020). The supply side of the model requires data on the techno-economic parameters of the power plants (capacity, efficiency, fuel shares, capital cost, and O&M costs) as well as fuel costs (Lund and Thellufsen, 2020). For VRES generation, the model requires the hourly distribution of the VRES. In EnergyPLAN, conventional power plants are grouped as combined heat and power (CHP) and power plant (PP) units. Other parameters needed for the analysis include the interest rate and environmental emission factors. The output of EnergyPLAN is the energy balances which encompass primary energy supply, final energy demand, electricity import/export, CO_2_ emissions, and annualized energy system costs.

EnergyPLAN has been widely used for energy system analysis to aid the design of sustainable energy systems (Østergaard, 2015). Besides national energy system pathways analyses, EnergyPLAN has been used to investigate the role of certain technologies in energy transition such as bioenergy (Kwon and Østergaard, 2013), hydropower (Askeland et al., 2019), desalination (Østergaard et al., 2014), compressed energy storage (Lund and Salgi, 2009) and many other studies pertaining to 100% renewables system (Hansen et al., 2019). Given the versatility and transparency of EnergyPLAN model, its results can be easily compared with other studies. Here, we apply EnergyPLAN towards modeling EVs interaction with the power sector and investigate positive interaction that may arise with VRES.

#### Transport energy demand, electricity demand, and electricity supply

The Nigerian energy system has been characterized in terms of transportation energy demand as well as electricity supply and demand with available 2015 data. In 2015, the share of EVs in Nigeria's transportation sector was almost zero (in our analysis, we assumed zero EVs), and the sector was entirely powered by fossil fuels (diesel and gasoline) (Dioha et al., 2021). Nigeria's transportation energy demand has been modeled with reference to the African Energy Commission's energy database for the year 2015 (AFREC, 2021). Due to Nigeria-specific data paucity, the Italian transport hourly distribution has been adopted for this study (Heat Roadmap Europe, 2016). However, this does not impact the analysis substantially as most transport distributions are similar - having morning and evening rush hours (Heat Roadmap Europe, 2016). Moreover, model results are meant to be qualitative and directional, and caution should be applied to avoid over-interpretation of precise numerical results.

EnergyPLAN model structure requires data input for the transportation sector's total gasoline, diesel, and electricity consumption. However, our analysis is focused on light-duty vehicles (LDVs) and not on all vehicles. Fuel consumption of heavy-duty vehicles also contributes to the total final energy consumption of the sector. However, Nigeria's energy balances do not distinguish gasoline and diesel consumption according to the types of vehicles (AFREC, 2021). Consequently, to derive the fuel (gasoline and diesel) consumption of the LDVs, we subtracted their total fuel consumption from the total fuel consumption of the Nigerian transportation sector. In 2015, the demand for gasoline and diesel for transportation was around 88.9 and 6.2 TWh, representing around 93% and 7% of the sector's final energy consumption (AFREC, 2021).

Data gaps plague estimation of Nigeria’s LDVs fuel-wise consumption. There are no consistent statistical records of different types of LDVs’ gasoline and diesel consumption. Different approaches to modeling transportation sector energy demand have been established. A detailed description of these approaches is given in (Rezvany et al., 2021). Transport energy consumption is a function of the total population, vehicle technology, fuel choice, total distance traveled, mode of travel, driving style, and vehicle occupancy (Anable et al., 2012). To determine the fuel consumption of the different LDVs, an activity-based bottom-up approach was used (Rezvany et al., 2021).

The road transportation sector is disaggregated into vehicle-wise passenger-kilometer demand in this study. The objective here is to separately estimate the passenger travel demand for each type of LDVs. The LDVs are divided into four categories:

• motorcycles (including two- and three-wheelers)
• private cars
• light commercial vehicles, LCVs (including taxis and *danfos*)
• light buses (urban bus).

The energy consumption of the LDVs has been derived according to Equation (1) (TERI, 2006):

(Equation 1) $$ED_{LDV,2015}=\sum_{t}\left(V_{t}\times O_{t}\times U_{t}÷FE_{t}\right)$$

Where $ED_{LDV}$ is the total energy demand of the different types of LDVs (Petajoule – PJ), $V_{t}$ the total number of LDVs of type t, $O_{t}$ the load factor (the average number of passengers per vehicle type), $U_{t}$ the average annual kilometre (km) traveled by passengers in the different types of LDVs, and $FE_{t}$ the passenger fuel economy of the different types of LDVs (billion passenger-km per Petajoule – BPkm/PJ). EneryPLAN requires input energy data in the Terawatt hour (TWh) unit. Therefore, a conversion factor of 1 PJ = 0.277778 TWh was used.

The total number of the different types of LDVs (motorcycle, private car, LCV, and light bus) in Nigeria in 2015 has been calculated from a World Bank study on low carbon development for Nigeria (Cervigni et al., 2013) by dividing the sum of the 2010 and 2020 LDV estimates by two as per Table 1.

It was assumed that 100% of the motorcycle run on gasoline and 2% of the private car, LCV, and light bus run-on diesel.

The average number of passengers per vehicle (load factor) is adapted from (Cervigni et al., 2013; UITP/UATP, 2010). The average annual kilometers traveled by the different types of LDVs are derived from (Cervigni et al., 2013), while fuel economies are adapted from (Dioha and Kumar, 2020a). It is worth mentioning that the fuel economies from (Dioha and Kumar, 2020a) were calculated at the activity level (BPkm/PJ) and not at the vehicular level. This has been adopted to suit our analysis due to the lack of country-specific data for vehicular fuel economies across the different road transportation modes. Table 2 presents the 2015 values of vehicle population, load factor, annual utilization factor, and fuel economy.

There is huge uncertainty with respect to Nigeria's electricity demand as it is split between the centralized and the off-grid power system (Tambari et al., 2020). As earlier noted, we assume the charging demand of EVs will be served solely by the centralized-grid electricity system. We use the term “centralized-grid” to distinguish from isolated mini-grids. There are no reliable estimates for Nigeria's centralized-grid annual electricity demand; however, Roche et al. (2020) estimated this value to be ∼26.0 TWh. On the other hand, the IEA energy balances for Nigeria showed an estimate of 27.6 TWh (IEA, 2021). This study uses a conservative value of 25.09 TWh, as reported by the African Energy Commission for 2015 (AFREC, 2021). The electricity hourly distribution profile has been derived from (Adeoye and Spataru, 2019; Toktarova et al., 2019).

The central electricity supply system (e.g., generation capacities, type of power plants) has been modeled according to (Roche et al., 2020). In 2015, Nigeria's central electricity system comprised natural gas power plants (5.845 GW) and large hydro (1.383 GW). For future scenarios, we consider the addition of solar and wind to the existing central electricity generation mix. Solar and wind hourly distribution profiles (capacity factors) are calculated based on the Modern-Era Retrospective analysis for Research and Application, Version-2 (MERRA-2) reanalysis product. For solar, we assume a single-axis tracking solar panel system (north-south direction), with a tilt of the solar panel to be 0° and a maximum turning angle of 45°. For the wind turbine, we assume a wind turbine hub height of 100 m. The wind capacity factor calculation adopts a piecewise function consisting of four parts delineated by a cut-in speed, a maximum rated power speed, and a cut-out speed.

#### Costs and emission factors

The fuel costs have been obtained from (Dioha and Kumar, 2020b) and they are presented in Table 3.

Table 4 shows the capital cost of the different LDVs considered. Due to the variation of vehicle cost in the country, we used different car buying websites in Nigeria (including https://www.ccarprice.com/ng/, https://carsforsalenigeria.com/, https://naijauto.com/, https://autochek.africa/ng, and https://carmart.ng/) as well as foreign costs to calculate the average capital cost of each type of LDV. A conversion factor of NGN 1 = US$400 was used. The capital cost refers to the fixed, one-time expenses incurred on the purchase of a vehicle. This cost does not include the infrastructure cost needed to provide service stations for the vehicles (see limitations of the study).

Table 5 outlines the techno-economic parameters used in modeling the existing and future electricity generating technologies. These parameters are adapted from (Nigerian Economic Summit Group and Heinrich Böll Stiftung Nigeria, 2017).

An interest rate of 3% was assumed for all cost categories (Bamisile et al., 2021). The emission factors for oil (gasoline & diesel) and gas are taken as 74.0 and 56.7 kgCO_2_/GJ (0.20412 and 0.2664 kgCO_2_/kWh), respectively.

#### Modeling EVs in EnergyPLAN

We have modeled the charging strategy of EVs in three different ways.

(i) normal cars
(ii) flexible cars, and
(iii) Vehicle-to-Grid (V2G) cars

Normal cars are modeled to be charged based on the charging habits of the vehicle owners without any form of regulation or consideration of the balance of electricity supply or demand. The flexible cars are modeled to charge only when there is excess electricity production from VRES to avoid grid overloading, but they do not inject power into the grid. The V2G cars are charged during excess electricity production and inject power into the grid (i.e., having a bidirectional flow of power).

In EnergyPLAN, a series of input data is used to model EVs as per EnergyPLAN's simulation logic. The model requires the composite annual electricity demand of the EVs and the hourly transport distribution profile, which it uses to calculate the number of V2G EVs that are driving and thus not connected to the grid at the hour in question. The hourly distribution profile alongside the maximum fraction of V2G EVs driving during peak demand hour(s) is also used to calculate the share of the V2G EVs available to the power system in any given hour. Additionally, the hourly distribution of transport demand is used to calculate the discharging of the battery when the vehicle is in operation. It is worth noting that all parameters used to define the V2G system are for the whole vehicle fleet within the region in question. For example, the batteries of the vehicles involved in V2G are modeled as a single big battery for the entire fleet.

The hourly EV (transportation) demand and thereby discharging of the battery storage is calculated as per Equation (2).

(Equation 2) $$t_{V2G}=\left(D_{V2G}\times \frac{\delta_{V2G}}{\sum \delta_{V2G}}\right)\;\times \eta_{CHARGE}$$

where $t_{V2G}$ represent the discharging of the battery, $D_{V2G}$ the electricity demand of the vehicles participating in V2G (TWh/year), $\delta_{V2G}$ the annual hourly distribution of the transportation demand, and $\eta_{CHARGE}\;$ the efficiency of discharging of battery.

The grid connection capacity of the entire V2G portion of the fleet $\left(c_{V2G}\right)$ on an hourly basis is determined according to Equation (3):

(Equation 3) $$c_{V2G}=C_{Charger}\times V2G_{Connection-Share}\times \left(\left(1-V2G_{Max.\;Share}\right)+V2G_{Max.\;Share}\times \left(1-\delta_{V2G}/Max\left(\delta_{V2G}\right)\right)\right)$$

Equation (3) consists of three parameters. The first parameter is $C_{Charger}$, the power capacity of the whole fleet participating in V2G (MW). This is multiplied by $V2G_{Connection-Share}$ the share of parked vehicles plugged into the grid. The third parameter, in the bracket, calculates the fractional share of on-road vehicles at each hour. The third factor in the bracket is based on the sum of two terms: the first term, $\left(1-V2G_{Max.\;Share}\right)$, corresponds to the minimum share of parked vehicles, while the second term, $\left(1-\delta_{V2G}/Max(\delta_{V2G}\right)$, represents the additional share of vehicles parked during non-rush hours. The fraction of vehicles parked at each hour is determined from the transportation hourly demand profile. This equation consequently yields $c_{V2G}$, the power capacity of all connected V2G fleet, in any given hour.

The grid connection capacity of the entire V2G depends on the definition of the maximum V2G vehicles, $V2G_{Max.\;Share}$, and $V2G_{Connection-Share}$. Practically, it is assumed that vehicles only charge and discharge to the grid when they are parked. By setting a value for $V2G_{Max.\;Share}$, we can define how many vehicles are parked and potentially connected to the grid during peak demand. In this study, the $V2G_{Max.\;Share}$ has been taken as 20% – indicating a typical case where at least 80% of all vehicles are parked during rush hours (Kempton et al., 2001). Accordingly, we assume that the share of parked vehicles plugged into the grid $\left(V2G_{Connection-Share}\right)$ is 70% (Lund and Thellufsen, 2020). The grid connection charging and discharging capacity of individual motorcycle, private car, LCV and light bus is taken as 4.0, 10.0, 10.0 and 11.5 kW, respectively.

In EnergyPLAN, V2G EVs will charge when there is critical excess electricity production $\left(e_{CEEP}\right)$ as well as available battery capacity $\left(S_{V2G-Battery}-\;s_{V2G-Battery}\right)$ within the limitation of the electrical capacity of the grid ($c_{V2G}$) for a given hour. Equation (4) is the minimum of the three values:

(Equation 4) $$e_{Charge}=min\;\left(e_{CEEP,}\left(S_{V2G-Battery}-\;s_{V2G-Battery}\right)/\mu_{Charge,\;}\;c_{V2G}\right)$$

where $e_{Charge}$ represent the battery charging, and $\mu_{Charge\;}$ the efficiency of the charger which is taken as 90% (Perujo and Ciuffo, 2010).

As noted above, EV charging is induced in a situation where current transportation demand and the next "y" hours cannot be delivered by the stored battery energy. In EnergyPLAN, the "y" value is initially set to 1 h. However, if this results in a lack of battery content, the value is increased in steps of 1 h.

The minimum battery content required $\left(S_{V2G-Battery-min}\right)$ is determined as per Equation (5):

(Equation 5) $$S_{V2G-Battery-min}=\sum_{a+y}^{x=a}t_{V2G}$$

Next, the battery charging is modified accordingly, requiring that (Equation 6):

(Equation 6) $$e_{Charge}\geq \left[S_{V2G-battery}-S_{V2G-battery-min}\;\right]/\mu_{Charge\;}$$

When $e_{Charge}$ is greater than the capacity of the grid connection, then $\;c_{V2G}$, the number of hours, "y", is increased by one, and the calculations are repeated. Therefore, the new battery content is calculated by adding the above charging and subtracting the discharging induced by driving $\left(t_{V2G}\right)$ according to Equation (7):

(Equation 7) $$s_{V2G-Battery:}:=s_{V2G-Battery}-t_{V2G}+\left(e_{Charge}\times \mu_{Charge\;}\right)$$

The V2G EVs are simulated to supply electricity to the grid in a scenario where there is a potential replacement of production from power plants $\left(e_{PP}\right)$ after satisfying transportation demand (Equation 8):

(Equation 8) $$e_{Inv}=min\;\left[e_{PP,}\;\left(\left(s_{V2G-Battery}-\;s_{V2G-Battery-min}\right)\times \mu_{Inv}\right),\;c_{V2G}\right]$$

where $e_{Inv}$ represent supply to the grid, $e_{PP}$ the power plants, and $\mu_{Inv}$ the efficiency of the inverter (battery-to-grid connection taken as 90%).

Consequently, the new battery content is determined as per Equation (9):

(Equation 9) $$s_{V2G-Battery:}:=s_{V2G-Battery}-\left(e_{Inv}/\mu_{Inv\;}\right)$$

All the equations and procedures for V2G modeling implemented in EnergyPLAN are fully explained in (Lund and Kempton, 2008), while the full description of the EnergyPLAN modeling tool is given in (Lund et al., 2021; Lund and Thellufsen, 2020)*.*

#### Experiment

As earlier mentioned, the purpose of this study is not to forecast EV sales or Nigeria’|'s energy system evolution but to provide an additional context for informed discussions. Accordingly, different EV penetration levels and VRES capacities have been modeled for the Nigerian energy system while keeping 2015 electricity demand levels and conventional power plants unchanged. We assume that the load profile, driving characteristics, and total LDVs remain the same as in 2015. However, as VRES capacity is progressively added to the system, power production from conventional fossil power plants is reduced as EnergyPLAN prioritizes power generation from VRES. Similarly, as we progressively replace conventional LDVs with EVs, the fuel consumption of the transportation sector is gradually shifted from fossil fuel (gasoline and diesel) to electricity.

To construct future scenarios for our study, we combine the different levels of VRES capacities and the EV penetration levels to understand their positive interaction within an integrated energy system framework.

For future VRES capacities, solar PV and wind capacity have been parametrically increased from 0 GW in 2015 to reach maximum capacities of 20 GW for solar PV and 4 GW for wind power. Table 6 shows the different VRES capacities considered while constructing our scenarios. It may be observed that solar PV is prioritized ahead of wind power due to the abundance of solar resources in the country as opposed to wind resources. Nigeria has enormous solar energy potential with average solar radiation of 3.5–7.0 kWh/m^2^/day (Dioha and Kumar, 2018; ECN, 2014; Tijjani et al., 2017), while its wind regime is low to moderate with an average annual wind speed of 3.5–5.5 m/s (Dioha et al., 2016). It is worth noting that the capacity values used here are for experimental purposes only, as they could possess different values in real-life development.

As earlier noted, our focus is on light-duty vehicles (LDVs). Consequently, to determine future scenarios for the transportation sector, we linearly decrease gasoline- and diesel-based LDVs and simultaneously replace them with EVs until they are fully replaced while still keeping the total LDVs number at the 2015 level. Table 7 presents the total energy consumption of the LDVs at different EV penetration levels.

The combination of Tables 6 and 7 yielded 55 scenarios as described in Table 8.

We analyzed the implication in terms of curtailment (excess electricity production), CO_2_ emission, and total annual energy system cost under different charging strategies (i.e., normal, flexible and V2G) for each scenario.

## Data Availability

• Data: All data reported in this paper will be shared by the lead contact upon request. • Code: This paper does not report original code. The EnergyPLAN model employed in this paper is written in Delphi Pascal, and a detailed description of the model's algorithms is available from www.energyplan.eu. • Any additional information required to reanalyze the data reported in this paper is available from the lead contact upon request.
